# A Comprehensive Robust Adaptive Controller for Gust Load Alleviation

**DOI:** 10.1155/2014/609027

**Published:** 2014-02-04

**Authors:** Elisa Capello, Giorgio Guglieri, Fulvia Quagliotti

**Affiliations:** Politecnico di Torino, Dipartimento di Ingegneria Meccanica ed Aerospaziale, Corso Duca degli Abruzzi 24, 10129 Turin, Italy

## Abstract

The objective of this paper is the implementation and validation of an adaptive controller for aircraft gust load alleviation. The contribution of this paper is the design of a robust controller that guarantees the reduction of the gust loads, even when the nominal conditions change. Some preliminary results are presented, considering the symmetric aileron deflection as control device. The proposed approach is validated on subsonic transport aircraft for different mass and flight conditions. Moreover, if the controller parameters are tuned for a specific gust model, even if the gust frequency changes, no parameter retuning is required.

## 1. Introduction

Active control techniques for the reduction of airframe gust loads are usually studied and designed to improve passenger comfort and to control the aeroelastic response improving the aircraft handling qualities. Different approaches have been investigated in the last years, including design of classical robust controllers, such as the linear quadratic regulator theory [1, 2], optimal control algorithms [3], and *H*_*∞*_ robust control [4, 5]. In the work of Aouf et al. [6], five flexible modes are considered in the aircraft dynamic model and the gust signals are assumed to be generated by the Dryden power spectral density model. In this research, the gust power is concentrated in the frequency band (0.1–6) Hz. The first problem of this *H*_*∞*_ controller is that the performance is guaranteed only if the model represents the aircraft dynamics perfectly; this means that no uncertainties or variations in the nominal system parameters are considered. More recently, Jansson and Eller [5] have implemented a robust controller including uncertainties in the model parameters to ensure stability and sufficient performance even in the presence of errors in the nominal model. Only the wing bending (difference of deformation at the wing tip and the wing root) is considered as flexible variable. One drawback of this controller is that the order of magnitude of the gust action is about twenty seconds; thus, it is not realistic. Moreover, the results obtained in this paper are not exhaustive enough because the magnitude of the rigid variables is increased for the uncertain system. The wing bending is reduced by about 20% but only for a particular choice of perturbation; that is, the gust is included as a Gaussian white noise of unit intensity and zero mean.

A limitation of the classical robust controller is that it is difficult to synthesize a *unique* control law for the whole flight envelope and gain scheduling should be required to account for the time varying characteristics of the aircraft dynamics. For this reason, adaptive feedback and/or feedforward controllers have been considered for adverse situations due to their ability to modify a preexisting control design. Zeng et al. [7] proposed an adaptive controller feedforward controller in which the aircraft configuration changes are taken into account via a real time system identification algorithm. A limitation of this controller is the application of a Single Input-Single Output (SISO) problem; this means that only one variable can be alleviated with the controller. Wildschek [8] proposed an adaptive Multiinput-Multi output (MIMO) feedforward controller and a feedback *H*_2_ controller. No uncertainties are considered and the adaptive controller alleviated only the wing bending acceleration.

The objective of the present paper is to derive a *unique* controller, robust in the presence of model uncertainties due to weight and flight condition variations and able to guarantee a stable aeroelastic response with gust load alleviation. Moreover, we aim at proposing an industrial application of *ℒ*_1_ adaptive control techniques [9] in the framework of flexible fixed wing subsonic aircraft, designing a controller able to stabilize the system under different operating conditions and when different gusts occur. A complete flexible model for the longitudinal plane is analyzed, including linearized equations of motion written in modal coordinates. Unsteady aerodynamic forces are synthesized by Padè approximants.

The paper is organized as follows. In Section 2, the model description and formulation are presented. In the same section, the gust and actuator models are described. In Section 3, the control architecture of the *ℒ*_1_ controller is introduced. The controller design and the simulation results are described in Section 4. Conclusions are summarized in Section 5.

## 2. Model Description

The longitudinal plane of a regional aircraft is considered for the validation of the adaptive controller for gust load reduction. The mathematical formulation of the dynamic system is modeled with standard continuous time-invariant state space formulation:

(1) x˙(t)=Ax(t)+Bu(t)+Bgwg(t), x(t)∈Rn,  u(t),wg(t)∈Rm,y(t)=Cx(t)+Dgwg(t),  x(0)=x0,   y(t)∈Rl,

where *x*(*t*) is the state vector, *u*(*t*) is the control signal, *y*(*t*) is the controlled output, and *w*_*g*_(*t*) is the gust signal. *A* is the state matrix, *B* is the input matrix, *C* is the output matrix, *B*_*g*_ is the input gust matrix, and *D*_*g*_ is the output gust matrix. This complete aeroservoelastic model is obtained joining two sub-models: (i) the flight dynamic model, that describes the rigid body motion of the aircraft, and (ii) the aeroelastic model, which is responsible for the aircraft aeroelasticity. Hypothesis of small disturbances from a steady flight condition allows linearizing the rigid body equations of motion [10] and uncoupling the longitudinal plane response from the lateral one. The rigid state variables are the longitudinal component of the total airspeed *u*, the angle of attack *α*, the pitch angle *θ*, and the pitch rate *q*. The mass and the elastic properties of the aircraft are given by a beam model made in Nastran and the flexible formulation can be obtained from the classical formulation of motion equations for multidegree-of-freedom systems, as in

(2) $$\begin{array}{c} M\ddot{x}\left(t\right)+C_{k}\dot{x}\left(t\right)+Kx\left(t\right)=F\left(t\right), \end{array}$$

where *M* is the mass matrix, *C*_*k*_ is the viscous damping matrix, and *K* is the stiffness matrix. The flexible vector *x*(*t*) is the general time varying displacement vector and *F*(*t*) can be divided into two terms, one related to the aerodynamic components induced by the structural normal modes and the second related to external forces that may not be depending on aerodynamics. The control devices are the elevator surface *δ*_*e*_ and the (outboard and inboard) aileron surfaces *δ*_*a*_ou__ and *δ*_*a*_in__. For both devices, only their symmetric deflection is considered.

The external aerodynamic force is modelled considering unsteady aerodynamics and is introduced in the statespace model (1) using Padè interpolation method [11]. The aerodynamic forces *F*_*t*_ and the gust forces *F*_*g*_ can be modeled as

(3) $$\begin{array}{c} F_{t}\left(s\right)=\frac{1}{2}\rho V_{\text{tas}}^{2}\text{FGT}\left(s\right)\delta \left(s\right),\\ F_{g}\left(s\right)=\frac{1}{2}\rho V_{\text{tas}}^{2}\text{FGM}\left(s\right)w\left(s\right), \end{array}$$

where *V*_tas_ is the aircraft true airspeed, *δ*_*i*_ is the control device deflection (*i* = *e*, *a*_ou_, *a*_in_), *w* is the gust speed, and FGT(*s*) and FGM(*s*) are large-scale improper transfer function matrices of the particular forms

(4) $$\begin{array}{c} \text{FGT}\left(s\right)=F_{t0}+F_{t_{1s}}+F_{t_{2s}}s^{2}+\overset{N}{\underset{i=1}{\sum }}F_{i+2}\frac{s}{s+\sigma_{i}},\\ \text{FGM}\left(s\right)=F_{g0}+F_{g_{1s}}+F_{g_{2s}}s^{2}+\overset{N}{\underset{i=1}{\sum }}F_{gi+2}\frac{s}{s+\sigma_{gi}} \end{array}$$

with *F*_∗_ and *σ*_∗_ being interventing coefficient matrices and filter poles from FEM analysis [12].

As far as a unique way to define delay terms will not be established, a possible way to obtain reliable and accurate results is to perform sensitivity tests on delay sets and to choose delay sets based on the better matching of interpolating curves with original data.

### 2.1. Models of the Gust Input and Loads

Time domain aeroelastic analysis is performed to generate responses to active control and/or to external force systems. Gust input causes a variation of the system aerodynamics, that is, simulated in state space formulation using the Doublet-Lattice Method [13]. Panel incidence induced by the gust is computed for each control point of the aerodynamic mesh, that has to be introduced in the model in terms of *x*, *y* and *z* coordinates. The generation of the induced angle of attack due to the gust profile (*F*_gust_*j*__(*t*)) for each aerodynamic control point is expressed in (5) (discrete gust 1 − cosine model for a reference system with *z* upward and *x* backward),

(5) $$\begin{array}{c} F_{\text{gus}{\text{t}}_{j}}\left(t\right)=\frac{U}{2U_{\infty }}\cos\left(\gamma_{j}\right)\left[1-\cos\;\left(2\pi f_{g}\left(t-\frac{x_{0}-x_{j}}{U_{\infty }}\right)\right)\right], \end{array}$$

where *U* is the vertical gust speed, *U*_*∞*_ = *V*_tas_ is the aircraft airspeed, cos⁡(*γ*_*j*_) is the dihedral cosine of each panel control point, and *f*_*g*_ is the gust frequency. The distance between the aircraft reference system center and the gust is defined as *x*_0_ = *d*_*g*_ *U*_*∞*_, with *d*_*g*_ = 0.1 s gust time delay. The variable *x*_*j*_ is the *x* coordinate of the *j*th aerodynamic control point.

The gust dynamic loads have been calculated by means of the mode displacement (MD) method, which recovers the loads directly from the modal displacements. The MD approach assumes that the modal superposition assumption, used to construct the generalized aeroelastic equations of motion, can also be used to recover the load distributions. Since gust excitation cases are characterized by fairly well-distributed loads, the MD method can be successfully used to calculate the actual loads with a sufficient number of modes. The modal superposition assumption is

(6) $$\begin{array}{c} \xi \left(x,t\right)=\phi \left(x\right)\eta \left(t\right), \end{array}$$

where *ϕ*(*x*) is the matrix of modal displacements and *η*(*t*) is the vector of natural modes in the range of 1 to 50 Hz of frequency. This assumption implies that the aerodynamic and inertial modal load (forces and moments), integrated for obtaining section loads, can be expressed as

(7) $$\begin{array}{c} F\left(x,t\right)=C_{\text{LOAD}}\eta \left(t\right), \end{array}$$

where *C*_LOAD_ is the integrated stiffness matrix expressed in modal form.

### 2.2. Actuator Model

The simulation model includes an electrohydroStatic actuator (EHSA) coupled with its related aileron surface and it contains the following nonlinearities:

- input command saturation and computational delay,
- rate saturation and output surface position saturation (implemented inside the control loop).

For the validation of the control laws, the maximum aileron rate is imposed equal to 80 deg/s.

## 3. *ℒ*_1_ Adaptive Controller

**Figure 1:**
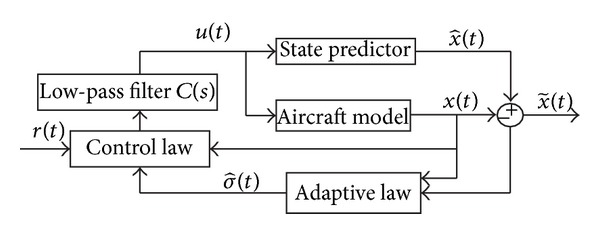
*ℒ*_1_ controller architecture.

The choice of the *ℒ*_1_ adaptive controller for the aircraft control is motivated by the high level of model uncertainty and by the variations of the mass and flight conditions. The *ℒ*_1_ adaptive controller here applied, extensively described in [9], takes into account unmatched uncertainties which include unmodeled dynamics and state- and time-dependent nonlinearities. This controller is composed by three main blocks: (i) the adaptive law, (ii) the state predictor, and (iii) the control law. See Figure 1 for the detailed architecture.

The adaptive law is a piecewise constant law, as explained in Chapter 3.3 of [9] and in [14], that guarantees fast estimation, and the adaptation rate can be associated with the sampling rate of the onboard CPU. Moreover, this adaptive algorithm guarantees bounded inputs and outputs, uniform transient response, and steady-state tracking. This extension of the *ℒ*_1_ controller was applied to NASA's AirSTAR [15] and to the Boeing X-48B [16].

The state predictor, which is designed to reproduce the actual plant structure and to specify the desired behavior of the closed loop system, generates a prediction of the system state. This prediction, when subtracted from the actual system state, yields an error signal that, together with the measured state signal and the control signal, drives the adaptation process. An important feature of the *ℒ*_1_ controller is that the error between the closed loop system with the *ℒ*_1_ controller and the reference controller can be uniformly bounded by a constant proportional to the adaptation sampling rate.

Another important key aspect is that this controller defines the control signal as the output of a low-pass filter to guarantee that the control signal stays in the low-frequency range. The filter is introduced with the understanding that uncertainties in any feedback loop can only be compensated for within the bandwidth of the control channel. The low-pass filter for this application is designed with a mixed deterministic and randomized approach as described in [17].

The above described controller is designed to control the general linear system of (1) which, considering uncertainties, can be rewritten as

(8) x˙(t)=Amx(t)+Bm(ωu(t)+f1(x(t),z(t),t)) +Bumf2(x(t),z(t),t),   x(0)=x0,z(t)=g0(xz(t),t),x˙z(t)=g(xz(t),x(t),t),  xz(0)=xz0,y(t)=Cx(t).

The matrix *A*_*m*_ ∈ *R* ^*n*×*n*^ is Hurwitz and specifies the desired dynamics of the closed loop system and *B*_*m*_ ∈ *R* ^*n*×*m*^ and *C* ∈ *R* ^*m*×*n*^ are known constant matrices. *B*_*um*_ ∈ *R* ^*n*×(*n*−*m*)^ is a constant matrix such that *B*_*m*_^*T*^ *B*_*um*_ = 0 and the rank of *B* = [*B*_*m*_ *B*_*um*_] is *n*. Compared to system (1), the system (8) includes *ω* ∈ *R* ^*m*×*m*^ the unknown frequency gain matrix, *z*(*t*) and *x*_*z*_(*t*), respectively, the output and state vector of internal unmodeled dynamics and the unknown nonlinear functions *g*(·) and *g*_0_(·).

The unknown nonlinear functions *f*_1_(·) and *f*_2_(·) satisfy the condition

(9) [f1(x(t),z(t),t)f2(x(t),z(t),t)]=B−1f(x(t),z(t),t).

The state predictor is defined as

(10) x^˙(t)=Amx^(t)+Bm(ω0u(t)+σ^1(t))   +Bumσ^2(t), x^(0)=x0,

where the adaptive vectors ${\hat{\sigma }}_{1}(t)\in R^{m}$ and ${\hat{\sigma }}_{2}(t)\in R^{n-m}$, with *ω*_0_ a candidate nominal frequency, are

(11) $$\begin{array}{c} \left[\begin{array}{c} {\hat{\sigma }}_{1}\left(t\right)\\ {\hat{\sigma }}_{2}\left(t\right) \end{array}\right]=-\left[\begin{array}{cc} {𝕀}_{m} & 0\\ 0 & {𝕀}_{n-m} \end{array}\right]B^{-1}\Phi^{-1}\left(T_{s}\right)\mu \left(iT_{s}\right), \end{array}$$

for *i* = 0,1, 2,…, and *t* ∈ [*iT*_*s*_, (*i* + 1)*T*_*s*_], where *T*_*s*_ > 0 is the adaptation sampling time associated with the sampling rate of the FCS computer. In (11) also appear

(12) $$\begin{array}{c} \begin{array}{cc} & \Phi \left(T_{s}\right)=A_{m}^{-1}\left(e^{A_{m}T_{s}}-{𝕀}_{n}\right),\in R^{n\times n}\\ & \mu \left(iT_{s}\right)=e^{A_{m}T_{s}}\tilde{x}\left(iT_{s}\right), \end{array} \end{array}$$

where $\tilde{x}(t)=\hat{x}(t)-x(t)$ is the error between the system state and the predicted state.

Finally, calling *s* the complex argument resulting from the Laplace transform of the corresponding time domain signal, the last element of the controller is the control law defined as

(13) $$\begin{array}{c} u\left(t\right)=-KD\left(s\right)\hat{\eta }\left(s\right). \end{array}$$

We also define

(14) $$\begin{array}{c} \hat{\eta }\left(t\right)=\omega_{0}u\left(t\right)+{\hat{\eta }}_{1}\left(t\right)+{\hat{\eta }}_{2m}\left(t\right)-r_{g}\left(t\right),\;\\ {\hat{\eta }}_{1}\left(t\right)={\hat{\sigma }}_{1}\left(t\right),\\ {\hat{\eta }}_{2m}\left(s\right)=H_{m}^{-1}\left(s\right)H_{um}\left(s\right){\hat{\sigma }}_{2}\left(s\right),\\ r_{g}\left(s\right)=K_{g}\left(s\right)r\left(s\right), \end{array}$$

where *D*(*s*) is a proper stable transfer matrix of dimension *m* × *m* and *r*(*t*) is the reference signal. The transfer functions *H*_*m*_ and *H*_*um*_ are calculated starting from the matrices of systems (8)

(15) $$\begin{array}{c} H_{m}\left(s\right)=C{\left(s{𝕀}_{n}-A_{m}\right)}^{-1}B_{m}, \end{array}$$

(16) $$\begin{array}{c} H_{um}\left(s\right)=C{\left(s{𝕀}_{n}-A_{m}\right)}^{-1}B_{um} \end{array}$$

while the prefilter *Kg*(*s*) is chosen as the constant matrix *K*_*g*_ = −(*CA*_*m*_^−1^ *B*_*m*_)^−1^ to achieve decoupling among the signals.

## 4. Controller Design and Simulation Results

The aircraft states are both rigid and flexible components, related to the aircraft longitudinal plane. Fourteen natural modes *η*_*i*_ in the range of frequency 1 to 50 Hz and three Padè terms for the definition of unsteady aerodynamic coefficients are considered. The sampling rate of the adaptive controller is equal to 100 Hz, a typical value for FCS computer. The input controls are the elevator and splitted aileron symmetric deflections. As controlled variables for gust alleviation, the dynamic loads in the wing root working station (WS21) have to be reduced to guarantee the controller efficiency, even when uncertainties occur (i.e., variation of the mass, flight condition and gust frequency). In addition, the rigid variables and the acceleration on the IMU station (near the aircraft centre of gravity) and right wing tip are evaluated. For the flexible variables, the vertical force and the moments around *X* and *Y* axes are analyzed, as the loads are directly evaluated from the natural modes (7). The aircraft characteristics are reported in Table 1.

Even if a complete rigid-flexible model is considered, the controller state predictor reproduces only the rigid dynamics and the flexible components are indirectly controlled by these variables. The scope of this simplified design is to verify whether or not the overall system can be controlled by using the rigid states of the aircraft [18].

For the design of the state predictor, the pole placement must be done considering the desired dynamic specifications, that is, fast response with high damping to prevent the gust peaks. To validate the results obtained with this controller (rigid predictor), a comparison with the open loop response is considered.

Different simulations are performed:

1. nominal case (see Table 1) with ideal and real actuator,
2. different mass and flight conditions,
3. variations of mass, flight conditions, and gust frequency.

**Figure 2:**
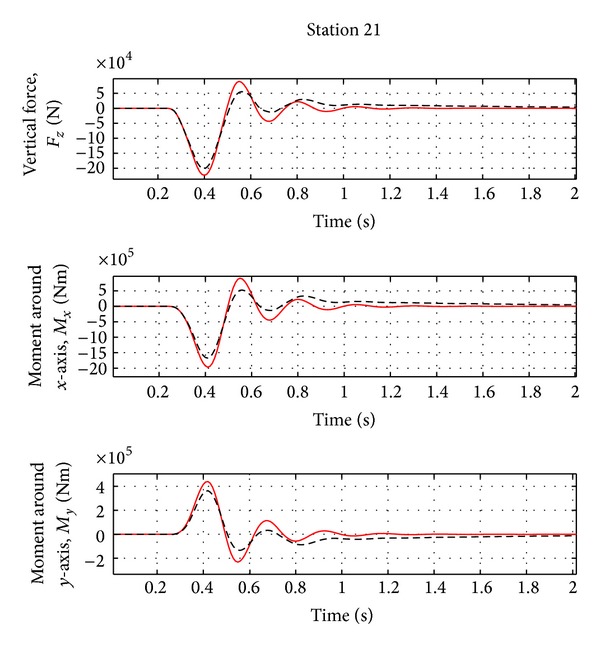
Wing loads for the nominal case (Case A, Table 3). Red solid line: open loop. Black dotted line: closed loop.

**Figure 3:**
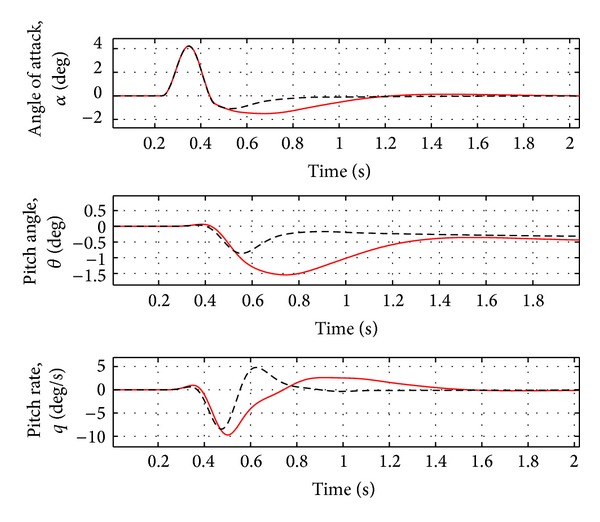
Rigid variable variation for nominal case (Case A, Table 3). Red solid line: open loop. Black dotted line: closed loop.

**Figure 4:**
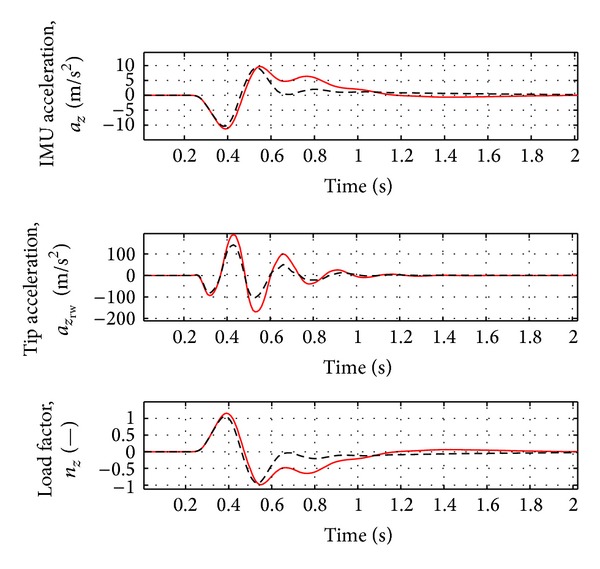
Variation of acceleration and load factor for nominal case (Case A, Table 3). Red solid line: open loop. Black dotted line: closed loop.

As evident from Figure 2, the first peak of the aircraft vertical force at wing root is reduced by about 10% and the first peak of the moments around *X* and *Y* axes is reduced by about 15–17%. As evident from Figure 3, a damped response of the rigid variables is guaranteed and the steady state error is less than 2%. The acceleration at the IMU station and at the right wing tip is reduced by about 5–10%, as in Figure 4. See Table 2 for the results.

### 4.1. Ideal and Real Actuator

To verify the effectiveness of the control laws, only the aircraft model is considered, without any real actuator models. Some simulations are performed: (i) without any actuator model (ideal actuator with no delay and bandwidth of more than 100 Hz) and (ii) EHSA actuator model (see Section 2.2). A maximum aileron deflection of ±15 deg and a maximum aileron rate of 80 deg/s are imposed for both cases.

**Figure 5:**
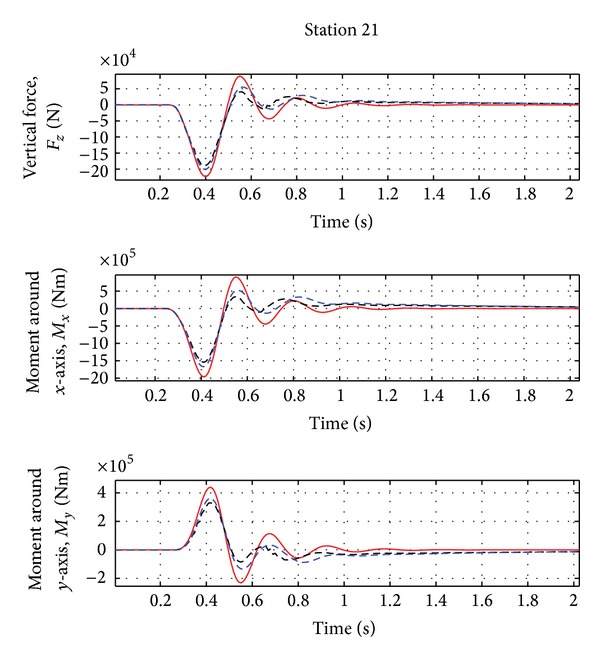
Wing loads for the nominal case with ideal and real actuator. Red solid line: open loop. Black dotted line: ideal actuator model. Blue dotted line: real actuator model.

As visible in Figure 5, a reduction of the vertical force *F*_*z*_ of 16% is obtained with an ideal actuator, even considering a limitation on the aileron rate. Better results are obtained for the moments around *X* and *Y* axes: more than 20% of reduction is obtained with an ideal actuator model.

**Figure 6:**
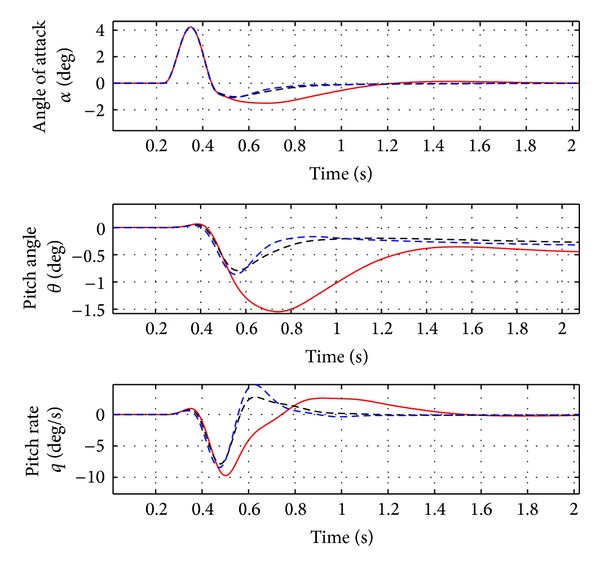
Rigid variable variation for nominal case with ideal and real actuator. Red solid line: open loop. Black dotted line: ideal actuator model. Blue dotted line: real actuator model.

**Figure 7:**
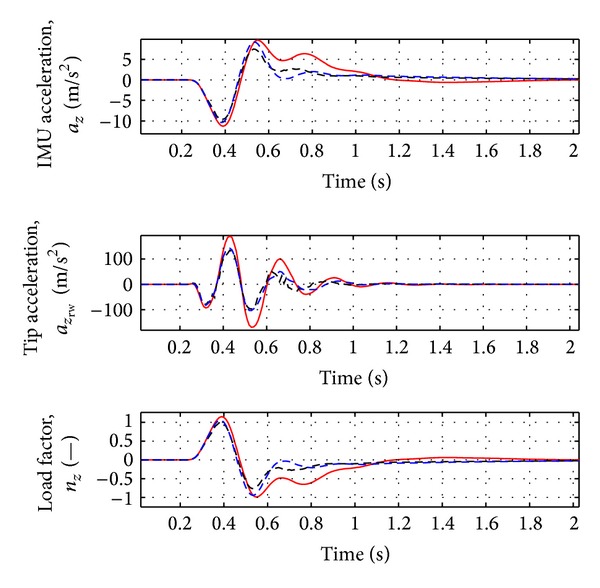
Variation of acceleration and load factor for nominal case with ideal and real actuator. Red solid line: open loop. Black dotted line: ideal actuator model. Blue dotted line: real actuator model.

In Figure 6 the variation of the rigid variables is reported. As for the nominal case, a damped response of these variables is guaranteed. The same behavior can be observed for the accelerations at IMU station and at the wing tip (see Figure 7).

### 4.2. Different Mass and Flight Conditions

**Figure 8:**
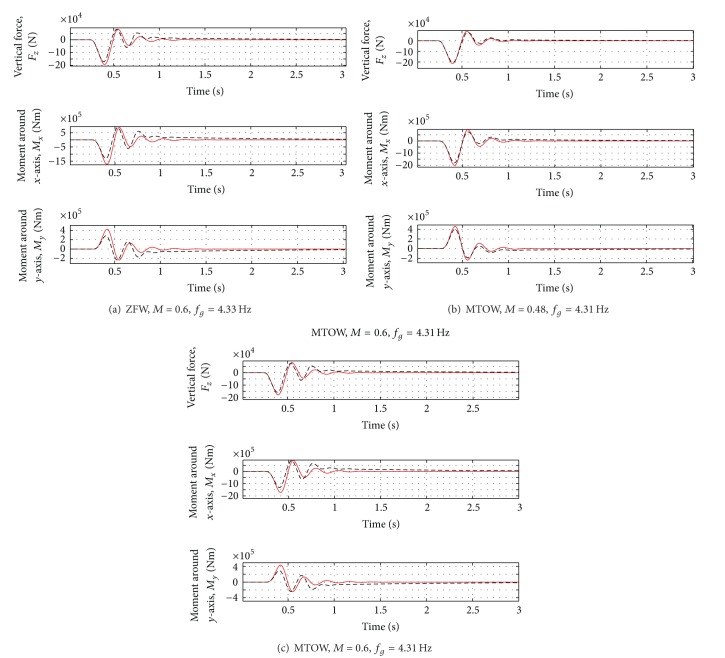
Wing loads for all the cases in Table 3. Red solid line: open loop. Black dotted line: closed loop.

**Figure 9:**
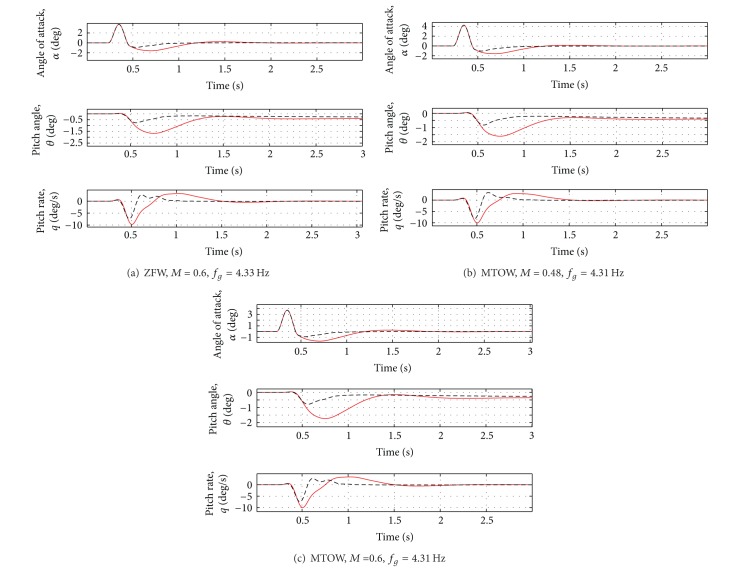
Rigid variable variation for all the cases in Table 3. Red solid line: open loop. Black dotted line: closed loop.

**Figure 10:**
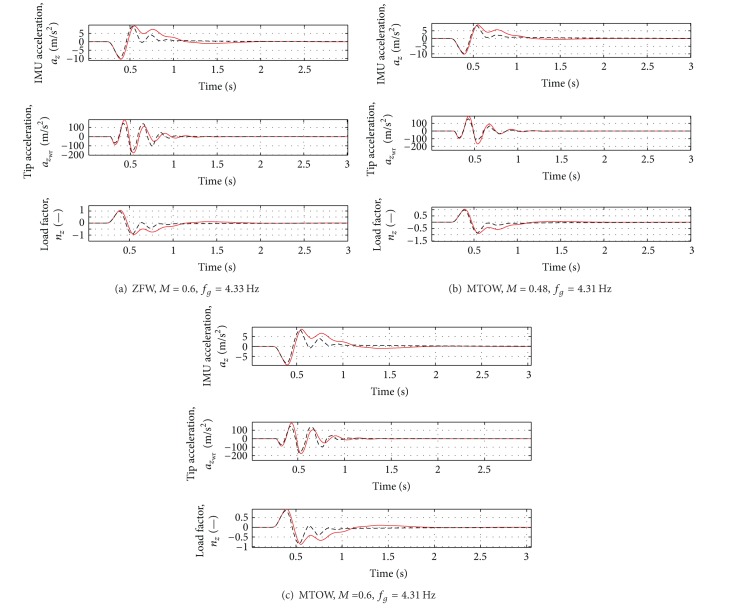
Variation of acceleration and load factor for all the cases in Table 3. Red solid line: open loop, Black dotted line: closed loop.

Variations on total airspeed (variations on Mach number and altitude) and on the aircraft mass (Zero Fuel Weight. ZFW and Maximum Take-Off Weight. MTOW) are considered. A combined variation of these variables is implemented to validate the adaptive controller. The same gust input signal previously analyzed (gust frequency *f*_*g*_ = 4.33 Hz for ZFW, *f*_*g*_ = 4.31 Hz for MTOW) and EHSA actuator model are considered. Case A is the nominal one (ZFW, *M* = 0.48, *f*_*g*_ = 4.33 Hz). The results are reported in Figures 8, 9 and 10. See Table 4 for the summary of the obtained results.

The airspeed and the aircraft mass are varied to take into account turbulence due to wind and changes in the payload mass. This validation is performed to verify that retuning of the controller parameters is not required if aircraft configuration changes are included in the complete model.

For classical robust controller, small oscillation of the matrix parameters is allowed without changing the controller parameters. Classical robust controllers (as Linear Quadratic Regulator controller) require retuning of the weight parameters if the model parameter variation is more than 15%, as explained in [19]. If an *ℒ*_1_ adaptive controller is implemented, the adaptation law permits following the desired responses without loss of robustness, due to the separation between the adaptation and the robustness (as in [9]).

**Figure 11:**
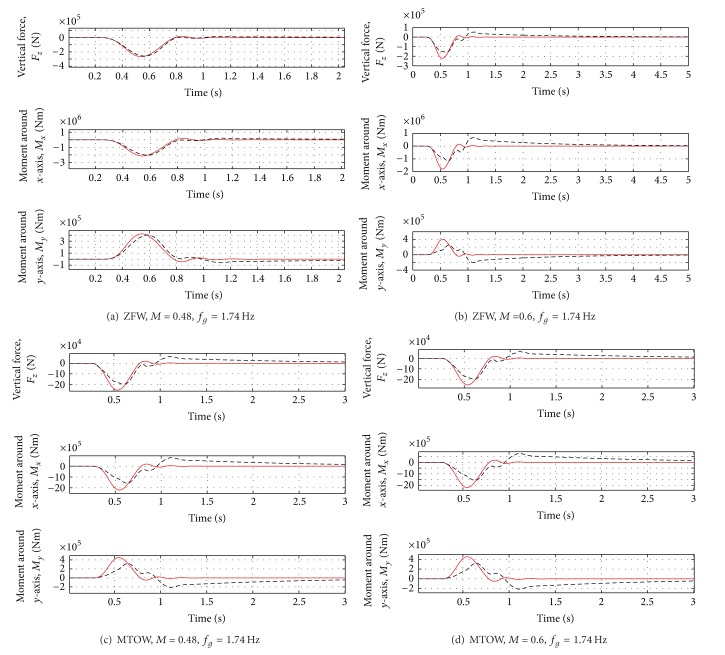
Wing loads for all the cases in Table 5. Red solid line: open loop. Black dotted line: closed loop.

**Figure 12:**
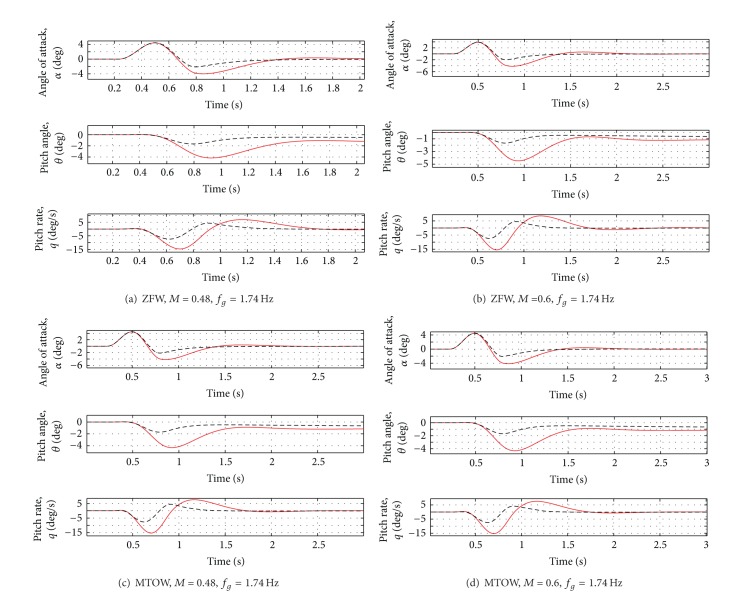
Rigid variable variation for all the cases in Table 5. Red solid line: open loop. Black dotted line: closed loop.

**Figure 13:**
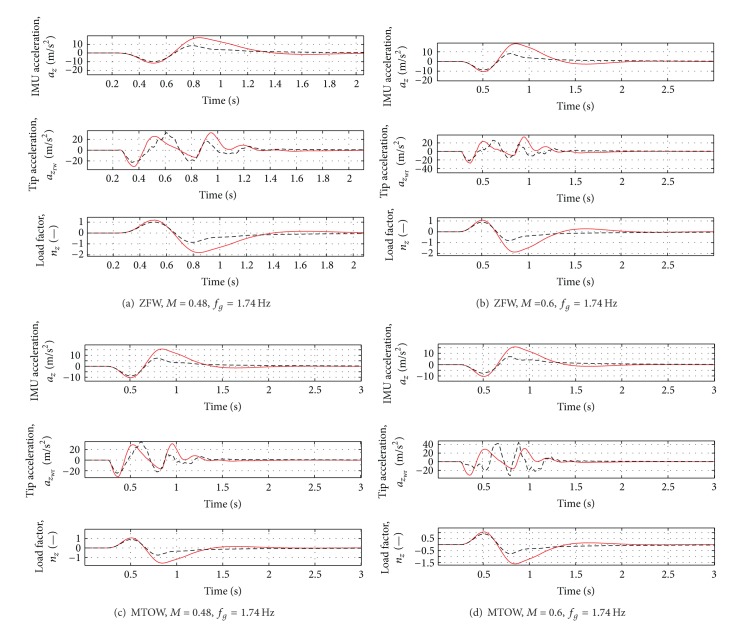
Variation of acceleration and load factor for all the cases in Table 5. Red solid line = Open Loop, Black dotted line = Closed loop.

Even in presence of variations of mass and flight conditions, the first peak of the vertical force is reduced by about 10% and the first peak of the moments by about 20–30%. For the most critical case (Case D of Table 3), a reduction of about 10% of all the loads can be observed. For the results, see Figures 11, 12, and 13.

### 4.3. Variations of the Gust Input

Adaptive control laws guarantee that if the system parameters are varied a retuning of the controller parameters is not required, as explained before. Usually, the adaptation channel is not useful when the disturbance input is changed. This means that, if different gust frequencies are considered, the controller tuned for a specific disturbance has to be changed. In our case we can verify that even if the controller parameters are tuned for a dynamic and fast gust (*f*_*g*_ = 4.33 Hz), retuning of the controller is not required for slower gust inputs, as a “quasi-static” gust (*f*_*g*_ = 1.74 Hz).

Even when the gust frequency is changed, the first load peak is reduced by about 20–30%. For the most critical case (Case H of Table 5), a reduction of about 25% of all variables is guaranteed. All the results are reported in Table 6.

## 5. Conclusions

Preliminary evaluation of the controller robustness in the presence of uncertainties is performed. The results are obtained with a sampling rate of 100 Hz, typical of FCS computer. Extensive simulation analysis is performed, including results with an ideal and a real actuator model. Good controller performance (alleviation of about 20% of the wing loads) is proved for different mass and flight condition configurations. In particular, a combined variation of flight parameters is taken into account. The complete system (controller and aircraft model) is also validated for a “quasi-static” gust input, to verify that a rigid predictor enforces alleviation of loads without requiring gain scheduling. As future development, load control will be performed with miniflaps as control devices.

## Figures and Tables

**Table 1:** Aircraft characteristics and flight conditions.

Mass	*m* = 50527 kg
Mean aerodynamic chord	*c* = 3.745 m
Wing span	*b* = 34.14 m
Vertical true airspeed	*V*_tas_ = 163.34 m/s
Mach number	*M* = 0.48
Gust frequency	*f*_*g*_ = 4.33 Hz

**Table 2:** Open and closed loop responses: ideal and real actuators.

Ideal actuator model			
Open loop	*F*_*z*_ = −2.235 · 10^5^	*M*_*x*_ = −1.965 · 10^6^	*M*_*x*_ = 4.402 · 10^5^
Closed loop	*F*_*z*_ = −1.887 · 10^5^	*M*_*x*_ = −1.545 · 10^6^	*M*_*x*_ = 3.301 · 10^5^
Real actuator model			
Open loop	*F*_*z*_ = −2.235 · 10^5^	*M*_*x*_ = −1.965 · 10^6^	*M*_*x*_ = 4.402 · 10^5^
Closed loop	*F*_*z*_ = −2.02 · 10^5^	*M*_*x*_ = −1.67 · 10^6^	*M*_*x*_ = 3.64 · 10^5^

**Table 3:** Analyzed cases: different mass and flight conditions.

Case A	*m* = 50527 kg	*M* = 0.48	*f*_*g*_ = 4.33 Hz
Case B	*m* = 50527 kg	*M* = 0.60	*f*_*g*_ = 4.33 Hz
Case C	*m* = 59000 kg	*M* = 0.48	*f*_*g*_ = 4.31 Hz
Case D	*m* = 59000 kg	*M* = 0.60	*f*_*g*_ = 4.31 Hz

**Table 4:** Open and closed loop responses for the cases of Table 3.

Case A			
Open loop	*F*_*z*_ = −2.235 · 10^5^	*M*_*x*_ = −1.965 · 10^6^	*M*_*x*_ = 4.402 · 10^5^
Closed loop	*F*_*z*_ = −2.02 · 10^5^	*M*_*x*_ = −1.67 · 10^6^	*M*_*x*_ = 3.64 · 10^5^
Case B			
Open loop	*F*_*z*_ = −1.928 · 10^5^	*M*_*x*_ = −1.716 · 10^6^	*M*_*x*_ = 4.402 · 10^5^
Closed loop	*F*_*z*_ = −1.711 · 10^5^	*M*_*x*_ = −1.309 · 10^6^	*M*_*x*_ = 2.83 · 10^5^
Case C			
Open loop	*F*_*z*_ = −1.788 · 10^5^	*M*_*x*_ = −1.725 · 10^6^	*M*_*x*_ = 4.343 · 10^5^
Closed loop	*F*_*z*_ = −1.581 · 10^5^	*M*_*x*_ = −1.321 · 10^6^	*M*_*x*_ = 2.912 · 10^5^
Case D			
Open loop	*F*_*z*_ = −2.688 · 10^5^	*M*_*x*_ = −2.134 · 10^6^	*M*_*x*_ = 4.223 · 10^5^
Closed loop	*F*_*z*_ = −2.593 · 10^5^	*M*_*x*_ = −2.026 · 10^6^	*M*_*x*_ = 3.991 · 10^5^

**Table 5:** Analyzed cases: different mass, flight conditions and gust frequency.

Case E	*m* = 50527 kg	*M* = 0.48	*f*_*g*_ = 1.74 Hz
Case F	*m* = 50527 kg	*M* = 0.60	*f*_*g*_ = 1.77 Hz
Case G	*m* = 59000 kg	*M* = 0.48	*f*_*g*_ = 1.74 Hz
Case H	*m* = 59000 kg	*M* = 0.60	*f*_*g*_ = 1.77 Hz

**Table 6:** Open and closed loop responses for all the cases of Table 5.

Case E			
Open Loop	*F*_*z*_ = −2.661 · 10^5^	*M*_*x*_ = −2.117 · 10^6^	*M*_*x*_ = 4.22 · 10^5^
Closed loop	*F*_*z*_ = −2.036 · 10^5^	*M*_*x*_ = −1.511 · 10^6^	*M*_*x*_ = 3.004 · 10^5^
Case F			
Open Loop	*F*_*z*_ = −2.214 · 10^5^	*M*_*x*_ = −1.795 · 10^6^	*M*_*x*_ = 4.401 · 10^5^
Closed loop	*F*_*z*_ = −1.518 · 10^5^	*M*_*x*_ = −1.148 · 10^6^	*M*_*x*_ = 2.539 · 10^5^
Case G			
Open Loop	*F*_*z*_ = −2.523 · 10^5^	*M*_*x*_ = −2.204 · 10^6^	*M*_*x*_ = 4.526 · 10^5^
Closed loop	*F*_*z*_ = −1.95 · 10^5^	*M*_*x*_ = −1.547 · 10^6^	*M*_*x*_ = 3.216 · 10^5^
Case H			
Open Loop	*F*_*z*_ = −2.535 · 10^5^	*M*_*x*_ = −2.203 · 10^6^	*M*_*x*_ = 4.502 · 10^5^
Closed loop	*F*_*z*_ = −1.935 · 10^5^	*M*_*x*_ = −1.547 · 10^6^	*M*_*x*_ = 3.209 · 10^5^

## References

[1] Fazelzadeh SA, Jafari SM (2008). Active control law design for flutter suppression and gust alleviation of a panel with piezoelectric actuators. *Smart Materials and Structures*.

[2] Patil MJ, Hodges DH (2002). Output feedback control of the nonlinear aeroelastic response of a slender wing. *Journal of Guidance, Control, and Dynamics*.

[3] Botez RM, Boustani I, Vayani N, Bigras P, Wong T (2001). Optimal control laws for gust alleviation. *Canadian Aeronautics and Space Journal*.

[4] Aouf N, Boulet B, Botez RM (2000). Robust gust load alleviation for a flexible aircraft. *Canadian Aeronautics and Space Journal*.

[5] Jansson N, Eller D Robust turbulence load alleviation.

[6] Aouf N, Boulet B, Botez R H2 and H_∞_-optimal gust load alleviation for a flexible aircraft.

[7] Zeng J, Moulin B, de Callafon R, Brenner MJ (2010). Adaptive feedforward control for gust load alleviation. *Journal of Guidance, Control, and Dynamics*.

[8] Wildschek A (2008). *An Adaptive Feed-Forward Controller for Active Wing Bending Vibration Alleviation on Large Transport Aircraft [Ph.D. thesis]*.

[9] Hovakimyan N, Cao C (2010). **ℒ*_1_ Adaptive Control Theory*.

[10] Etkin B, Reid L (1996). *Dynamics of Flight: Stability and Control*.

[11] Baker GA, Graves-Morris P (1996). *Padé Approximants*.

[12] Puyou G, Losser Y (2012). Clearance Benchmark for a civil aircraft. *Optimization Based Clearence of Flight Control Laws*.

[13] Rodden WP The development of the doublet-lattice method.

[14] Xargay E, Hovakimyan N, Cao C *ℒ*
_1_ adaptive controller for multi-input multi-output systems in the presence of nonlinear unmatched uncertainties.

[15] Hovakimyan N, Cao C, Kharisov E, Xargay E, Gregory IM (2011). *ℒ*
_1_ adaptive control for safety-critical systems. *IEEE Control Systems Magazine*.

[16] Leman T, Xargay E, Dullerud G, Hovakimyan N, Wendel T *ℒ*
_1_ adaptive control augmentation system for the X-48B aircraft.

[17] Capello E, Quagliotti F, Tempo R Randomized approaches and adaptive control for quadrotor UAVs.

[18] Avanzini G, Capello E, Piacenza I, Quagliotti F, Xargay E, Hovakimyan N *ℒ*
_1_ adaptive control of flexible aircraft: preliminary results.

[19] Polyak BT, Tempo R (2001). Probabilistic robust design with linear quadratic regulators. *Systems and Control Letters*.

